# Applying a One Health lens to understanding the impact of climate and environmental change on healthcare-associated infections

**DOI:** 10.1017/ash.2023.159

**Published:** 2023-05-19

**Authors:** Sabrina B. Graham, Catherine Machalaba, Sarah E. Baum, Jill Raufman, Sarah E. Hill

**Affiliations:** 1 Cornell University College of Veterinary Medicine, Ithaca, New York; 2 EcoHealth Alliance, New York, New York; 3 Global Health Center, Albert Einstein College of Medicine, Bronx, New York

## Abstract

The pace and trajectory of global and local environmental changes are jeopardizing our health in numerous ways, among them exacerbating the risk of disease emergence and spread in both the community and the healthcare setting via healthcare-associated infections (HAIs). Factors such as climate change, widespread land alteration, and biodiversity loss underlie changing human–animal–environment interactions that drive disease vectors, pathogen spillover, and cross-species transmission of zoonoses. Climate change–associated extreme weather events also threaten critical healthcare infrastructure, infection prevention and control (IPC) efforts, and treatment continuity, adding to stress to strained systems and creating new areas of vulnerability. These dynamics increase the likelihood of developing antimicrobial resistance (AMR), vulnerability to HAIs, and high-consequence hospital-based disease transmission. Using a One Health approach to both human and animal health systems, we can become climate smart by re-examining impacts on and relationships with the environment. We can then work collaboratively to reduce and respond to the growing threat and burden of infectious diseases.

Healthy, functioning ecosystems are essential to sustaining human health and survival. The current trajectory of anthropogenic environmental change puts our health at risk: widespread land alteration, biodiversity loss, pollution, and climate change.^1^ One way these changes affect our health is by exacerbating the risk of disease outbreaks as well as the emergence and spread of antimicrobial-resistant (AMR) pathogens in healthcare settings resulting in healthcare-associated infections (HAIs).^2^ It is crucial to define the effects of environmental change on increasing HAIs in order to propose solutions for reducing the threat of these diseases.

## Direct environmental drivers of HAI

The emergence and re-emergence of zoonotic and vectorborne diseases can occur via pathogen spillover between animals and humans.^3^ Environmental change occurs as the result of actions such as deforestation and urbanization, in which human–animal–environment interactions can result in disease outbreaks. Cross-species transmission of zoonoses can increase the incidence of HAI when human–human spread occurs. There are ∼2 billion cases of zoonotic disease annually, resulting in >2 million deaths, and >700,000 global deaths each year are due to vectorborne diseases.^4^ Both zoonotic and vectorborne diseases have led to recent epidemics and pandemics such as severe acute respiratory syndrome (SARS), Ebola virus, plague, COVID-19, and Zika virus. Environmental contamination has also been linked to antibiotic use in healthcare and food production and to drug-resistant infections. Healthcare facilities are opportune sites for these outbreaks to spread, particularly in the absence of sufficient infection prevention and control (IPC) infrastructure.

Drastically shifting temperatures and weather patterns are examples of climate change that can create more favorable conditions for the expansion of vectors such as mosquitoes. Malaria and dengue are recognized as climate-sensitive diseases with increasing distribution and incidence in tropical climates. Further, many tropical regions may have fewer economic resources to support a robust healthcare infrastructure, which can lead to greater vulnerability for transmission in healthcare facilities.^5^ A rise in malaria can increase HAI risk through organ and tissue transplantation from infected donors in susceptible healthcare facilities.^6^ The wealthiest nations are disproportionately responsible for producing carbon emissions, yet the poorest countries face the greatest burdens of climate change.^7^ A health-equity perspective is crucial for understanding and addressing the consequences of climate change.

Pathogen spillover occurs when there is direct transmission between animals and people in regions where it may or may not have occurred previously.^8^ Intensification of agriculture, expansion of animals and livestock into previously untouched natural areas, and urbanization are shaping human–animal–environment interactions and increasing the likelihood of pathogen spillover. These human-driven behaviors contribute to environmental degradation that increases the risk of not only disease emergence but also potential outbreaks. Farmers, veterinarians, and others who interact with animals are particularly at risk where biosecurity practices and protocols are weak. Zoonotic pathogens of high concern, such as Nipah, Lassa fever, and coronaviruses, all have epidemic potential and the risk for high-consequence illness in humans via nosocomial transmission. Nipah virus may be transmitted to humans that have close contact with animals such as fruit bats, pigs, and horses. Those who are occupationally or otherwise exposed to these animals may be at higher risk for infection.^9^ Subsequently, those who become infected may seek care in a traditional healthcare setting, where they may introduce highly infectious pathogens into healthcare facilities. Other patients in these settings then become vulnerable to these pathogens and other secondary HAIs. Human behaviors that degrade the environment drive a chain of events and consequences that can result in increased HAIs.

## Indirect environmental drivers of HAI

Flooding and extreme weather events are further consequences of climate change that result in damage to critical healthcare infrastructure. These natural disasters interrupt reliable access to and provision of care and IPC measures. Extensive flooding can jeopardize sanitation protocols and lead to hospital contamination, which was observed during Hurricane Katrina in New Orleans where methicillin-resistant *Staphylococcus aureus* (MRSA) and *Salmonella* infections increased.^10^

Environmental changes, including increasing global temperatures, can also increase the prevalence of infections, which may result in greater strain on healthcare systems. In the summers of 2021 and 2022, heat waves across the globe exacerbated the effects of the COVID-19 pandemic. High healthcare utilizers and vulnerable groups, such as the elderly and chronically ill, were dually at risk of heat-related health problems and COVID-19 in healthcare settings. Additionally, healthcare workers required to wear personal protective equipment were at a greater risk for heat-related illnesses.^11^ Rising temperatures are a major component of climate change that adds pressure to healthcare facilities not only to contend with the exacerbation of patients’ underlying conditions but also to protect patients and staff from the direct consequences of extreme temperatures.

## One Health solutions

One Health proposes a collaborative approach to harness information, expertise, and resources across sectors to better understand and address risks and impacts to human, animal, and environmental health (Box 1). A One Health approach can strengthen health security by improving our ability to monitor pathogens across species, detect and address changes to our ecosystems and behavioral practices to reduce the risks of pathogen spillover and disease emergence, and build comprehensive early warning and outbreak response systems.

Box 1. One Health is an integrated, unifying approach that aims to sustainably balance and optimize the health of people, animals, and ecosystems. It recognizes the health of humans, domestic and wild animals, plants, and the wider environment (including ecosystems) are closely linked and interdependent. The approach mobilizes multiple sectors, disciplines, and communities at varying levels of society to work together to foster well-being and tackle threats to health and ecosystems, while addressing the collective need for clean water, energy and air, safe and nutritious food, taking action on climate change, and contributing to sustainable development.

*Working definition endorsed by the Food and Agriculture Organization of the United Nations, United Nations Environment Programme, World Health Organization, and World Organisation for Animal Health.*

IPC at healthcare facilities is critical to limiting the spread of infections in human populations, yet IPC strategies are often poorly resourced and operationalized. In low- and middle-income countries, an estimated 15% of patients acquire HAIs in the process of receiving care.^12^ With the growing AMR crisis, poor IPC puts everyone at risk.

Using a One Health approach, our health systems can become climate smart by re-examining their impact on and relationship with the environment around them. We must design healthcare infrastructure and systems that are resilient against natural disasters, and we must recognize and take action against our own role in contributing to climate change. Investments must also be made to increase the capacity of our healthcare workforce to efficiently manage zoonotic and vectorborne diseases.^13^

Humans represent just one side of the One Health triad, yet our choices also shape animal and ecosystem health outcomes. The healthcare sector is essential to sustaining human health, yet it contributes to anywhere from 1% to 5% of the global environmental footprint.^14^ Healthcare professionals have a vital role to play in calling attention to and addressing the urgent threat posed by human-driven environmental degradation. This can be accomplished in several ways:

- Consider animal and environmental exposures when investigating an illness.
- Recognize the impact of long-term and acute environmental changes on disease dynamics.
- Participate in primary prevention of pathogen spillover by empowering individuals and communities to engage in disease risk reduction practices.
- Advocate and take action to address the inequities and vulnerabilities that underlie health disparities and impede our ability to adapt to growing environmental challenges.
- Innovate change in the healthcare system to reduce our contribution to environmental impacts.
- Expand healthcare investments in resources and programs needed to protect animal and environmental health.

These applications of the One Health approach can help us address the climate and environmentally driven expansion of global disease threats. These actions will require us to take a multifaceted approach to addressing the expansion of infectious diseases, including HAIs, and to build stronger, more secure, and more equitable healthcare systems.
